# Suppression of *SlMBP15* Inhibits Plant Vegetative Growth and Delays Fruit Ripening in Tomato

**DOI:** 10.3389/fpls.2018.00938

**Published:** 2018-07-04

**Authors:** Wencheng Yin, Xiaohui Yu, Guoping Chen, Boyan Tang, Yunshu Wang, Changguang Liao, Yanjie Zhang, Zongli Hu

**Affiliations:** ^1^Laboratory of Molecular Biology of Tomato, Bioengineering College, Chongqing University, Chongqing, China; ^2^School of Life Sciences, Zhengzhou University, Zhengzhou, China

**Keywords:** *SlMBP15*, MADS-box, gibberellin, ripening, ethylene

## Abstract

MADS-box genes have been demonstrated to participate in a number of processes in tomato development, especially fruit ripening. In this study, we reported a novel MADS-box gene, *SlMBP15*, which is implicated in fruit ripening. Based on statistical analysis, the ripening time of *SlMBP15*-silenced tomato was delayed by 2–4 days compared with that of the wild-type (WT). The accumulation of carotenoids and biosynthesis of ethylene in fruits were decreased in *SlMBP15*-silenced tomato. Genes related to carotenoid and ethylene biosynthesis were greatly repressed. SlMBP15 can interact with RIN, a MADS-box regulator affecting the carotenoid accumulation and ethylene biosynthesis in tomato. In addition, *SlMBP15*-silenced tomato produced dark green leaves, and its plant height was reduced. The gibberellin (GA) content of transgenic plants was lower than that of the WT and GA biosynthesis genes were repressed. These results demonstrated that *SlMBP15* not only positively regulated tomato fruit ripening but also affected the morphogenesis of the vegetative organs.

## Introduction

MADS-box genes encode a group of important transcription factors that are widely distributed in plants, animals, and fungi (Messenguy and Dubois, 2003). In 1990, the studies on the MADS-box related to its essential role in floral organ development were published (Sommer et al., 1990; Yanofsky et al., 1990). Subsequently, the famous ABC model for floral organ construction was proposed based on extensive genetic and molecular studies (Coen and Meyerowitz, 1991). Improvements in this research introduced new regulators and extended the ABC model into the ABCDE model (Colombo et al., 1995; Pelaz et al., 2001). In *Arabidopsis*, the A-class genes include *APETALA1* (*AP1*) and *APETALA2* (*AP2*). The B-class genes contain *APETALA3* (*AP3*) and *PISTILLATA* (*PI*). The C- and D- class genes all belong to the AGAMOUS (AG) clade, while the E-class genes come from the SEPALLATA (SEP) clade. All these genes belong to the MADS-box except for *AP2* and its homologs (Theissen et al., 2000). So far, in tomato, the function of most of the genes contained in the ABCDE model has been well described. In addition, some other MADS-box genes that influence floral organ development have been identified. For example, our former study showed that the MADS-box gene *SlAGL6* affected the development of the sepal and petal (Yu et al., 2017). Overexpression of *SlFYFL* delayed sepal senescence, (Xie et al., 2014) while overexpressing the AGL15 clade gene *SlMBP11* delayed perianth senescence in tomato (Guo et al., 2017). Furthermore, *TM8* overexpression in tomato plants generated anomalous stamens with poorly viable pollen (Daminato et al., 2014). A recent study suggested the tomato MADS-box gene *SlMBP21* is a novel factor involved in organ size control (Li et al., 2017).

In addition to floral organ identity determination, MADS-box genes also take part in inflorescence development in tomato. The MADS-box gene *JOINTLESS* (*J*) suppresses sympodial meristem identity in inflorescence meristems (Szymkowiak and Irish, 2006). The AP1/FUL gene *MACROCALYX* (*MC*)-regulates inflorescence determinacy (Vrebalov et al., 2002). In addition, a lack of *MC* produced an indeterminate inflorescence in tomato (Nakano et al., 2012). A recent study revealed that *MC* exerts the function of regulating inflorescence development through an interaction with *SFT* and *J* (Yuste-Lisbona et al., 2016). Additionally, current research revealed three novel MADS-box genes that influenced inflorescence architecture (Soyk et al., 2017).

Tomato MADS-box genes are also involved in plant vegetative growth. Previous research showed that overexpression of *SlFYFL* delayed leaf senescence (Xie et al., 2014). The AP1/FUL transcription factor gene *MBP20* is involved in tomato leaf development, and overexpression of a dominant-negative form of *MBP20* led to leaf simplification and partly suppressed the increased leaf complexity of plants (Burko et al., 2013). Apart from tomato leaf development, MADS-box genes are also considered to affect tomato plant vegetative growth in other ways. For example, tomato plants with increased *SlMBP11* levels displayed reduced plant height, leaf size and internode length; highly branched growth from each leaf axil; and an increased number of nodes and leaves (Guo et al., 2017). In tomato, there are some studies on the regulation of abscission zone (AZ) formation by MADS-box family members. The deletion of the MADS-box gene *JOINTLESS* leads to the failure of activation of pedicel AZ development in tomato plants (Mao et al., 2000). Similarly, the *MC*-suppressed transformants produced by the introduction of an *MC* antisense construct displayed an absence of the AZ structure or a barely visible, knuckle-like structure. Further studies revealed that MC interacts physically with JOINTLESS and that the resulting heterodimer acquires a specific DNA-binding activity (Nakano et al., 2012). Subsequently, the SEP clade gene *SlMBP21* was identified as an additional transcription activator factor for the development of AZ in tomato and SlMBP21 may form higher-order protein complexes with JOINTLESS and MC (Liu et al., 2014).

It has been proved that MADS-box transcription factors are involved in abiotic stress response in some plant species such as *Arabidopsis* (Lee et al., 2005), rice (Lee et al., 2008, 2011) and wheat (Tardif et al., 2007). However, to date, only *SlMBP11* and *SlMBP8* have been reported to function in abiotic stress response. *SlMBP11* functions as a positive regulator in response to salt stress (Guo et al., 2016). Tomato plants with repressed FLC/MAF clade gene *SlMBP8* became more tolerant to drought and salt stress (Yin et al., 2017b).

In addition to the functions we described above, tomato MADS-box genes mediate the following processes: cuticle development (Gimenez et al., 2015; Garceau et al., 2017), parthenocarpy (Ampomah-Dwamena et al., 2002; Klap et al., 2016), fruit ripening and so on. Among these processes, the most notable role for MADS-box genes is regulating fruit ripening. In 2002, a tomato *ripening-inhibitor* (*rin*) mutant with fruits that failed to ripen and enlarged sepals was reported (Vrebalov et al., 2002). Gene repression and mutant complementation demonstrated that *RIN* regulates tomato fruit ripening (Vrebalov et al., 2002). However, a *RIN*-knockout mutation did not repress the initiation of ripening, and the fruits of the mutant showed moderate red coloring (Ito et al., 2017). Recent research re-evaluated the *rin* mutant and declared that *RIN* is not required for the initiation of ripening and the *rin* is not a null mutation but a gain-of-function mutation that produces a protein that actively represses fruit ripening (Ito et al., 2017). In addition to *RIN*, many other MADS-box genes were confirmed to participate in the regulation of tomato fruit ripening. Tomato plants with reduced *TAGL1* mRNA produced a fruit-maturation-inhibition phenotype with reduced carotenoids and ethylene (Vrebalov et al., 2009). Moreover, two tomato FRUITFULL (FUL) clade genes also play roles in fruit ripening. Dual suppression of *FUL1* (*TDR4*) and *FUL2* (*MBP7*) substantially inhibited fruit ripening by blocking ethylene biosynthesis and decreasing carotenoid accumulation (Wang et al., 2014). These two genes also regulate fruit ripening in an ethylene-independent way (Bemer et al., 2012). In our laboratory, some MADS-box genes regulating fruit ripening have been investigated to explore the mechanism of tomato ripening. In tomato, *SlMADS1* acts as a negative regulator of fruit ripening and regulates the expression of carotenoid and ethylene biosynthesis genes (Dong et al., 2013). Overexpression of the tomato MADS-box gene *SlFYFL* delayed fruit ripening by 3–5 days (Xie et al., 2014), and suppression of *SlMBP8* accelerated fruit ripening and induced the expression of carotenoid- and ethylene- biosynthesis genes (Yin et al., 2017a).

In previous research, we revealed the function of *SlMBP8*, one of the FLC/MAF clade members. However, the roles of the other members, *SlMBP15* and *SlMBP25*, in tomato are still unclear. Previous studies showed that *SlMBP15* and *SlMBP25* are expressed in both vegetative and floral tissues (Hileman et al., 2006), which indicates that they probably play important roles in various developmental processes. Here, we characterized *SlMBP15* (accession number: NCBI: XM_004252663 / SGN: Solyc12g087810.2; **Supplementary Table S1**), and the expression pattern analysis showed that *SlMBP15* accumulation in tomato fruits of different stages was greater than that in other organs, while in fruit-ripening mutants, *SlMBP15* was repressed on the whole. Therefore, we hypothesized that *SlMBP15* may modulate tomato fruit ripening. To confirm this, we generated transgenic tomato with *SlMBP15* repressed by RNAi (RNA interference). We show herein that the altered expression of SlMBP15 delayed fruit ripening providing strong evidence for our hypothesis. In addition, the morphology of the vegetative organs of the transgenic plants was different from that of the wild-type (WT).

## Materials and Methods

### Plant Materials and Growth Conditions

Tomato plants (S*olanum lycopersicon Mill. cv. Ailsa Craig AC^++^*) were used as the WT in this study. Transgenic and the wild-type plants were grown in the greenhouse conditions (16 h-day/8 h-night cycle, 25/18°C day/night temperature, 80% humidity, and 250 μmolm^-2^ s^-1^ light intensity). The experiments in this study were also repeated in our experimental farm in the natural condition. Two generations of the transgenic tomato plants were used in this study. Plants of the first generation (T_0_) came from the tissue culture, and the second generation (T_1_) from the seeds. For gene expression analysis, flowers were tagged at anthesis and the ripening stages of tomato fruits were divided according to the days post anthesis (dpa) and fruit color. In the wild-type, we defined the fruit of 20 dpa as IMG (Immature green) and 35 dpa as MG (Mature Green), which was characterized as being green and shiny with no obvious color change. The fruit with a color change from green to yellow was defined as B (Breaker) while B + 4 and B + 7 were fruits of 4 days after breaker and 7 days after breaker, respectively. Tomato pericarp of 0.5 cm wide along the equator was used as fruit sample. All the harvested samples were immediately frozen in liquid nitrogen and stored at -80°C. Three individual plants for each line were used for biological replicates in all the treatments.

### Isolation and Sequence Analysis of *SlMBP15*

Total RNA of all plant tissues was extracted from three biological replicates using the RNAiso Plus (Takara) according to the manufacturer’s instructions. Then 1 μg RNA was used to synthesize the first strand cDNA by reverse transcription polymerase chain reaction (M-MLV reverse transcriptase, Takara, China) with tailed oligo d(T)18 primer (5′ GCT GTC AAC GAT ACG CTA CGT AAC GGC ATG ACA GTG TTT TTT TTT TTT TTT TTT 3′). To clone the full-length of *SlMBP15* gene, the PCR was employed using primers *SlMBP15*-F (5′ GAA ATC CAG GTG GCA GAG CAC 3′) and *SlMBP15*-R (5′GCT GTC AAC GAT ACG CTA CGT AAC G 3′), and 1–2 μl cDNA was used as PCR template. The PCR reaction was performed using high fidelity Prime STARTMHS DNA polymerase (Takara) following the procedure below: 94°C for 5 min followed by 35 cycles of 30 s at 94°C, 30 s at 56°C and 30 s at 72°C, and a final extension at 72°C for 10 min. The amplified product was tailed using DNA A-Tailing kit (Takara) and linked to pMD18-T vector (Takara). Positive colonies were picked out via *Escherichia coli* JM109 transformation and confirmed by sequencing (BGI, China). The multiple sequence alignment of SlMBP15 with other MADS-box proteins was performed using DNAMAN 5.2.2 software. All accession numbers of proteins used in multiple sequence alignment and phylogenetic analysis were listed in **Supplementary Table S2**.

### Construction of the *SlMBP15* RNAi Vector and Plant Transformation

In order to repress the expression of the *SlMBP15* gene, a RNAi construct was assembled using the pBIN19 vector (Fray et al., 1994; Frisch et al., 1995). Firstly, the 320 bp *SlMBP15* specific DNA fragment used in the hairpin was amplified using the primers *SlMBP15*-F (5′ GAA ATC CAG GTG GCA GAG CAC 3′) and *SlMBP15*-R (5′ GGA AAG TCA GCG AAA TCC GTA 3′) tailed with *Kpn* I, *Cla* I and *Xho* I, *Xba* I restriction cutting site at the 5′ end, respectively. Secondly, the purified products were digested with *Xho* I/*Kpn* I and inserted into pHANNIBAL vector (Wesley et al., 2001) plasmid at *Xho* I/*Kpn* I restriction site in the sense orientation, while the products tailed with *Xba* I/*Cla* I restriction enzyme in the antisense orientation. Thirdly, the double-stranded RNA expression unit, containing the cauliflower mosaic virus (CaMV) *35S* promoter, *SlMBP15* fragment in the sense orientation, PDK intron, *SlMBP15* fragment in the antisense orientation and the OCS terminator was digested by *SacI*/*XbaI* and linked into plant binary vector pBIN19. At last, the generated binary plasmid was used for the transformation to tomato cotyledon explants by *Agrobacterium tumefaciens* (strain LBA4404) (Chen et al., 2004). The tissue culture plants were selected by kanamycin (80 mg l^-1^) and then analyzed by PCR with the primers NPTII-F (5′GAC AAT CGG CTG CTC TGA 3′) and NPTII-R (5′AAC TCC AGC ATG AGA TCC 3′) to determine the presence of T-DNA.

### Quantitative Real-Time PCR (qPCR) Analysis

Quantitative real-time PCR was performed using the SYBR^®^ Premix Ex Taq II kit (Takara, China) in a 10 μl total reaction volume (5.0 μl 2 × SYBR Premix Ex Taq, 1.0 μl primers, 1.0 μl cDNA, 3.0 μl ddH_2_O). All reactions were performed using a two-step method: 95°C for 30 s, followed by 40 cycles of 95°C for 5 s, and 60°C for 30 s. To remove the effect of genomic DNA and the template from environment, NTC (no template control) and NRT (no reverse transcription control) were performed. Additionally, three replications for each sample were used and the standard curves were run simultaneously. Tomato *CAC* gene was selected as internal standard (Exposito-Rodriguez et al., 2008). The primers *SlMBP15*-Q-F and *SlMBP15*-Q-R (**Supplementary Table S3**) were used to determine the expression levels of *SlMBP15* in wild-type, *Nr* (*never ripe*), *rin* (*ripening inhibitor*) and transgenic lines. The relative gene expression was calculated using the 2^-ΔΔ*C*^_T_ method (Livak and Schmittgen, 2001). All primers used for qPCR were listed in **Supplementary Table S3**.

### Measurement of Carotenoid Contents

Tomato carotenoid was extracted from the pericarp using a modified protocol from a previous report (Forth and Pyke, 2006). 1.0 g sample was milled into powder with liquid nitrogen. Then 10 ml of 60:40 (v/v) hexane-acetone was added and total carotenoids of the wild-type and RNAi lines fruits were extracted. The extract was centrifuged at 4000 *g* for 5 min and the absorbance of supernatant was measured at 450 nm. The carotenoid content was calculated with the following equations: total carotenoids (mg ml^-1^) = (OD_450_)/0.25. Three independent experiments were performed for each sample.

### Measurement of GA_3_ and Fruit Ethylene Release

The stem from 4-week-old tomato plants was collected and frozen in liquid nitrogen. GA_3_ was extracted and purified using GA_3_ kit (GA-4-Y Comin Biotechnology Co., Ltd., China) according to the manufacturer’s instructions. Then the concentration of endogenous bioactive gibberellin (GA_3_) were measured by HPLC (High Performance Liquid Chromatography).

The ethylene released by fruit was measured at B, B + 4 and B + 7 stages. Fruits were placed in airtight jars for 24 h at 28°C. 1 mL gas sample of the headspace was injected into a Hewlett-Packard 5890 series II gas chromatograph equipped with a flame ionization detector. The samples were compared with reagent grade ethylene standards of known concentration and normalized for fruit weight (Chung et al., 2010). Three biological replicates and three technical replications were adopted for the data analysis.

### Yeast Two-Hybrid Assay

The yeast two-hybrid assay was performed using the MATCHMAKER GAL4 Two-Hybrid System III according to the manufacturer’s protocol (Clontech). The ORF of SlMBP15 was amplified by PCR with the primer SlMBP15(Y)-F (5′ GGC GAA TTC GGG CTT TTA ATC GGC GAA AAA 3′) and SlMBP15(Y)-R (5′ CGC GGA TCC CGA CGA ATA CGA CGA TAA TCA 3′). The PCR product was digested using *Eco*RI and *Sal*I, and it was cloned into the pGBKT7 bait vector by the *Eco*RI/*Sal*I site. The recombined vector was named pGBKT7-SlMBP15 and transformed into yeast strain Y2HGold. The Y2HGold with bait was plated on SDO (SD medium -Trp) and TDO (SD medium -Trp, -His, and -adenine) to test the self-activation of pGBKT7-SlMBP15. In parallel, the ORF of SlMADS-RIN was amplified by primers SlRIN(Y)-F (5′CCG GAA TTC ATG GGT AGA GGG AAA GTA GA3′) and SlRIN(Y)-R (5′CGC GGA TCC TCA TAG ATG TTT ATT CAT3′). The product was cloned into the pGADT7 vector and transformed into yeast strain Y187. Subsequently, the Y2HGold with bait and the Y187 with prey were cultured together in 2 × YPDA medium for 24 h. Then, the culture was grown on DDO (SD medium lacking Trp and Leu) to screen the diploids containing prey and bait. After 2–5 days, fresh diploid cells were plated on QDO/X [SD medium lacking Trp, Leu, His, and adenine with X-a-Gal (5-bromo-4-chloro-3-indolyl-a-D-galactopyranoside)] to test if SlMBP15 interacts with SlMADS-RIN. The colonies developing blue color were picked out and cultivated again on plates with QDO/X medium. The plates were incubated at 30°C for 3–7 days. The empty prey and bait vectors were used as controls to remove false positive, respectively. At the same time, the positive control and negative control were cultured. The assay was repeated at least three times with fresh transformants.

### Statistical Analysis

The data in this study was presented as mean ± standard deviation. The significant difference between wild-type and transgenic lines was analyzed by Student’s *t*-test (*P* < 0.05). The measurement values came from the means of at least three biological replicates.

## Results

### Sequence and Expression Analysis of *SlMBP15*

**FIGURE 1:**
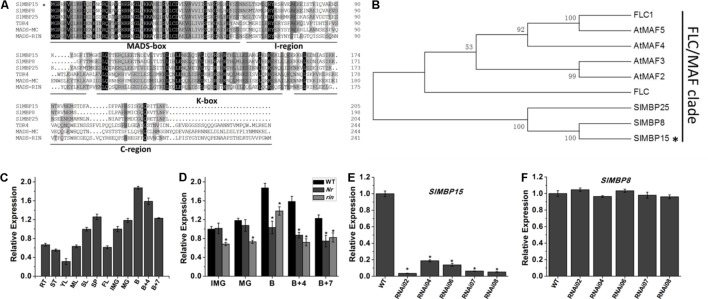
Sequence, phylogenic and expression analysis of *SlMBP15*. **(A)** Multiple sequence alignment of SlMBP15 with other MADS-proteins. **(B)** Phylogenic analysis of SlMBP15 and other MADS-box proteins. The phylogenic tree was constructed by the neighbor-joining method, bootstrap analysis of 1,000 replicates. SlMBP15 is marked with asterisk. Accession numbers of other proteins contained in phylogenic analysis are listed in **Supplementary Table S3**. **(C)** Expression profile of *SlMBP15* in WT. RT, root; ST, stem; YL, young leaf; ML, mature leaf; SL, senescent leaf; SP, sepal; FL, flower; IMG, immature green fruit; MG, mature green fruit; B, breaker fruit; B + 4, 4 days after breaker; B + 7, 7 days after breaker. **(D)** Expression pattern of *SlMBP15* in fruits of wild-type *Nr* and *rin* mutants. **(E)** Relative expression of *SlMBP15* in wild-type and transgenic tomato plants. **(F)** Relative expression of *SlMBP8* in wild-type and transgenic lines. Asterisks indicate a significant difference (*P* < 0.05).

We conducted the alignment with the gene sequence of *SlMBP15* by Basic Local Alignment Search Tool (BLAST)^1^. The result showed that there are three transcript variants (**Supplementary Table S4**). Even with some nucleotide difference, the transcript variant X3 and X4 translate the same protein. However, except for SlMBP15, the two proteins encoded by three transcript variants do not contain complete MADS-box domain (**Supplementary Figure S1**). We also carried out sequence alignment between SlMBP15 and other MADS-box proteins belonging to MIKC^C^ type using DNAMAN 5.2.2 software. The result displayed that SlMBP15 contained four typical domains (**Figure 1A**) which are shared by other MIKC^C^ proteins and the MADS-box domain was highly conserved. In addition, the phylogenic analysis showed that SlMBP15 belongs to FLC/MAF clade (**Figure 1B**).

In order to explore the expression profile of *SlMBP15*, we designed specific qPCR primers and detected the expression of *SlMBP15* in various tissues of wild-type tomato. Furthermore, the transcription of *SlMBP15* was also investigated in fruits at series developmental stages in wild-type, *Nr* and *rin* mutants. In wild-type tomato, the constitutive expression of *SlMBP15* indicated it may participate in the developmental processes in multiple tissues and organs of tomato (**Figure 1C**). In the wild-type tomato fruits, the expression of *SlMBP15* rose gradually along with the fruit development process, and the highest accumulation appeared at B stage. After that, *SlMBP15* was declined. The expression of *SlMBP15* decreased with the maturity of tomato fruit, which implied that *SlMBP15* may play important roles in fruit ripening. Therefore, we further compared the expression of *SlMBP15* in *Nr* and *rin* mutants with the wild-type. The transcript level of *SlMBP15* in *Nr* and *rin* was lower in fruits at almost all stages than that in the wild-type (**Figure 1D**), which suggested that *SlMBP15* may be regulated by *RIN* and *Nr*, and probably function downstream of these genes.

### Down-Regulation of *SlMBP15* Generated Dark Green Leaves in Tomato Plants

**FIGURE 2:**
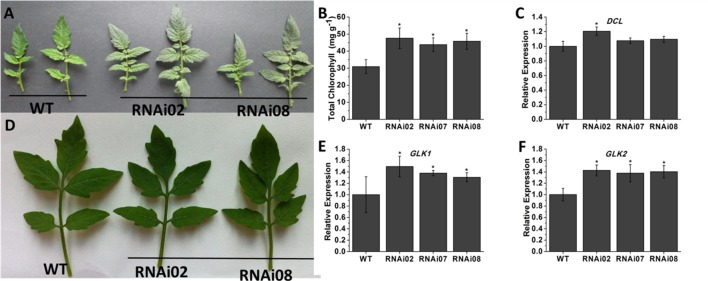
Phenotype of leaf color in *SlMBP15*-silenced lines. **(A)** The leaves detached from tomato plants grown in field. **(B)** The leaves detached from tomato plants grown in pots. **(C)** Total chlorophyll content in leaves. **(D–F)** Expression of genes related to chlorophyll biosynthesis. Each value represents the mean ± SE of three biological replicates. Asterisks indicate a significant difference (*P* < 0.05).

To explore the biological functions of *SlMBP15* in tomato, we generated transgenic tomato lines with the *SlMBP15* gene silenced. In total, we obtained 5 transgenic lines in which *SlMBP15* was well repressed, and the lines RNAi02, RNAi07 and RNAi08 were used for subsequent experiments because they showed the lowest expression levels of *SlMBP15* (**Figure 1E**). The expression of *SlMBP8*, the gene sequence shares the highest homology with *SlMBP15*, was not affected in the transgenic lines (**Figure 1F**). According to our observations, the compound leaves of transgenic plants were darker green in color than those of the wild-type both in field (**Figure 2A**) and in pots (**Figure 2D**). Then, we examined the total chlorophyll content, and the results demonstrated that the leaves of the three transgenic lines contained more chlorophyll than those of the wild-type, which was consistent with the previous observation (**Figure 2B**). Furthermore, we detected the transcript level of genes involved in the pathway of chlorophyll biosynthesis. As a result, a chloroplast development-related gene, *DCL* (*DEFECTIVE CHLOROPLASTS AND LEAVES*) (Keddie et al., 1996), and two photosystem-related genes *GLK1* (*Golden 2-like 1*) and *GLK2* (Powell et al., 2012), were examined. *GLK1* and *GLK2* determine the capacity for light-stimulated photosynthesis by controlling chloroplast formation (Powell et al., 2012). In tomato, co-suppression of *GLK1* resulted in pale leaves with reduced chlorophyll whereas co-suppression of *GLK2* resulted in fruits without a green shoulder (Nguyen et al., 2014). The tomato *dcl* mutation produces albino areas with abnormal palisade cells; moreover, the chloroplasts from proplastids are blocked (Keddie et al., 1996). qPCR results showed that *GLK1* and *GLK2* were significantly upregulated in the three transgenic lines, (**Figures 2E,F**) while *DCL* showed little change (**Figure 2C**).

### Silencing *SlMBP15* Inhibited Plant Growth and GA Biosynthesis

**FIGURE 3:**
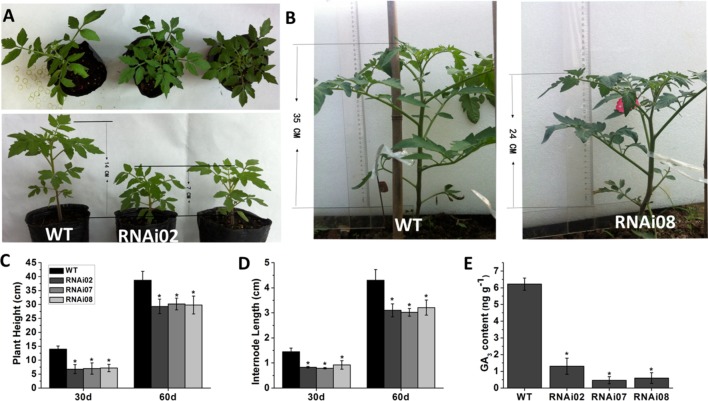
Phenotype of wild-type and transgenic plant stems. **(A)** Tomato plants of 30-day-old. **(B)** Tomato plants of 60-day-old. **(C)** Plant height of plants at 30 and 60-day-old, respectively. **(D)** The internode length of plants at 30 and 60-day-old, respectively. **(E)** The GA_3_ content of wild-type and transgenic lines.

In addition to having a different leaf color, the transgenic plants were shorter than the wild-type plants. We also measured the plant height and internode length of each transgenic line and the wild-type. On the 30th day from germination, the transgenic plants were 7 cm shorter than the wild-type (**Figures 3A,C**). A month later, the transgenic plants still remained 11 cm shorter than the wild-type plants (**Figures 3B,C**). The internode length of the transgenic lines was also shorter than that of the wild-type plants (**Figure 3D**).

**FIGURE 4:**
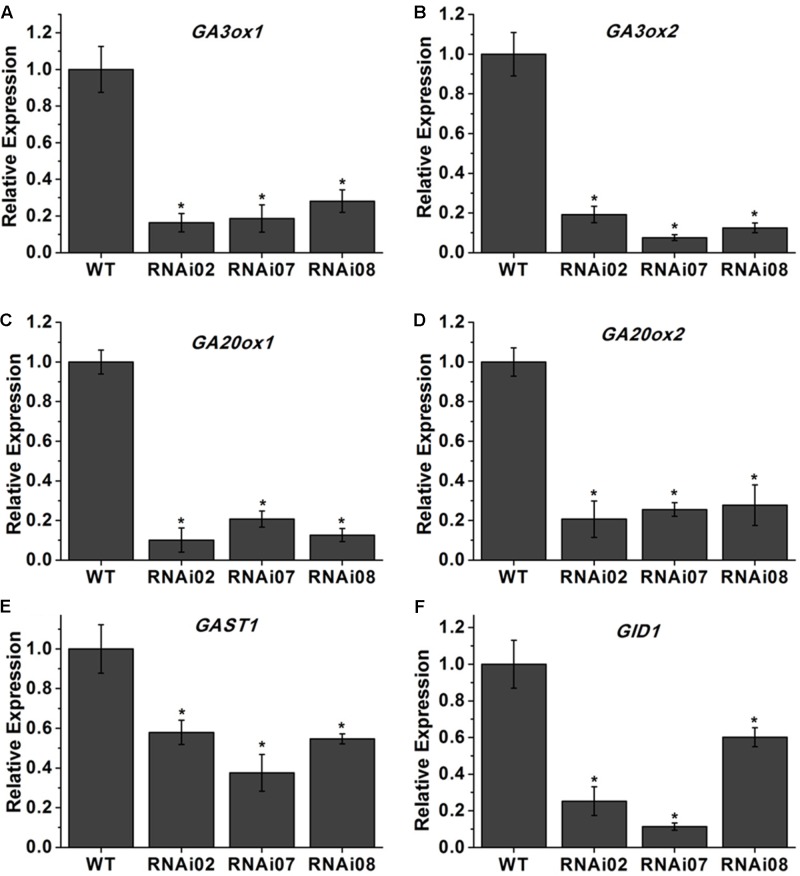
Relative expression of genes involved in GA biosynthesis. **(A–F)** The expression levels of *GA3ox1*, *GA3ox1*, *GA20ox1*, *GA20ox2*, *GAST1* and *GID1* in wild-type and transgenic lines. Each value represents the mean ± SE of three biological replicates. Asterisks indicate a significant difference (*P* < 0.05).

Previous research showed that GAs play an important role in stem elongation (Olszewski et al., 2002). Here, we measured GA_3_ content, and the result showed that the GA_3_ level in transgenic lines was significantly lower than that in the wild-type (**Figure 3E**). We further detected some genes known to be involved in GA biosynthesis. The GA20oxs act as important biosynthesis enzymes determining the GA concentration in many species, while the GA3oxs function in the final step of producing bioactive GAs (GA_1_, GA_3_, GA_4_, and GA_7_) (Yamaguchi, 2008). The transcription of *GAST1* (*tomato gibberellin-stimulated transcripts 1*) can be dramatically induced by GA (Shi and Olszewski, 1998). *GID2* (*GIBBERELLIN INSENSITIVE DWARF1*) is a receptor of GAs that is important for the perception and transduction of GA signaling (Ueguchi-Tanaka et al., 2007; Murase et al., 2008). In this study, the transcriptional level of all genes mentioned above was reduced (**Figure 4**). These results indicated that *SlMBP15* regulated the biosynthesis and response of GAs.

### Repression of *SlMBP15* Delayed Tomato Fruit Ripening

The downregulation of *SlMBP15* not only led to the difference in plant morphology at the vegetative growth stage but also inhibited tomato fruit ripening. To study the impact of *SlMBP15* on tomato fruit ripening, the number of days from anthesis to ripening was recorded. Statistical analysis of the data showed that the ripening time of the transgenic lines was delayed by 2–4 days compared with that of the wild-type (**Table 1**).

**Table 1:** Days from anthesis to breaker stage for wild-type and *SlMBP15*-silenced lines.

Tomato lines	Days
Wild-type	38.0 ± 0.50
RNAi02	42.6 ± 0.56^∗^
RNAi07	41.2 ± 0.38^∗^
RNAi08	40.4 ± 0.43^∗^


*Values represent means ± SD in days for at least 20 fruits of each line. Asterisks indicate a significant difference (*P* < 0.05).*

### Inhibition of *SlMBP15* Down-Regulated the Carotenoid Content

**FIGURE 5:**
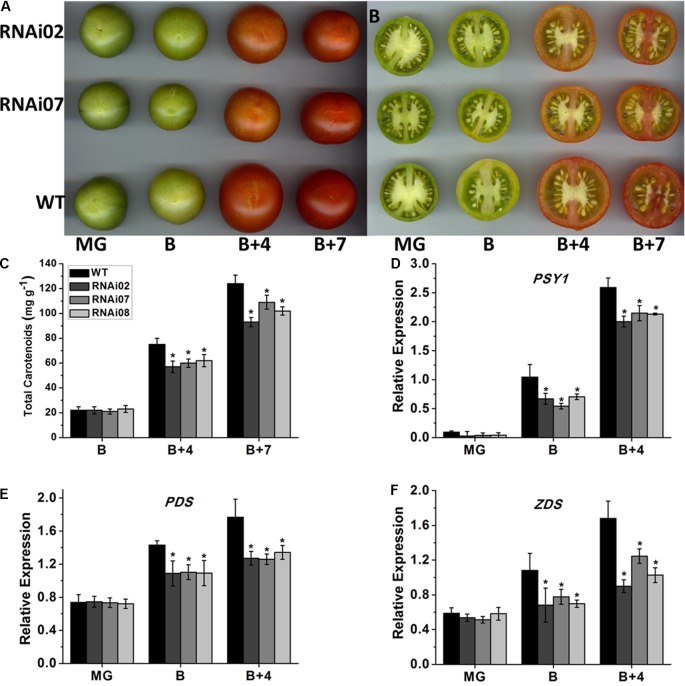
The accumulation of carotenoid in wild-type and transgenic lines. **(A,B)** The fruits of wild-type and transgenic lines at different stages. **(C)** The carotenoid content in fruits. **(D–F)** Relative expression of genes participating in carotenoid biosynthesis. Each value represents the mean ± SE of three biological replicates. Asterisks indicate a significant difference (*P* < 0.05).

Previous studies have demonstrated that tomato fruit ripening is accompanied by color alteration due to the continuous accumulation of carotenoids (Fraser et al., 1994). According to our observations, the pericarp color of *SlMBP15*-silenced lines at the B + 4 and B + 7 stages was lighter than that of the wild-type (**Figures 5A,B**). The test of carotenoid content in tomato pericarps demonstrated that, at the B + 4 and B + 7 stages, the tomato fruits of transgenic lines accumulated fewer carotenoids than those of the wild-type, which was in agreement with the lighter-color phenotype of transgenic tomato fruits (**Figure 5C**).

It is known that the biosynthesis of carotenoids is regulated by a group of genes, among which, PSY1 (phytoene synthase 1), PDS (phytoene desaturase) and ZDS (ζ-carotene desaturase) are important regulators (Hirschberg, 2001). To further confirm the underlying causes that led to the reduced accumulation of carotenoids in *SlMBP15*-silenced tomato fruits, the three genes were detected in tomato pericarps. qPCR analysis showed a remarkably lower expression level of *PSY1*, *PDS* and *ZDS* in the transgenic lines than in the wild-type at the B and B + 4 stages (**Figures 5D–F**). These results revealed that silencing *SlMBP15* inhibited the biosynthesis of carotenoids.

### Deficiency of *SlMBP15* Led to a Reduction in Ethylene Content

**FIGURE 6:**
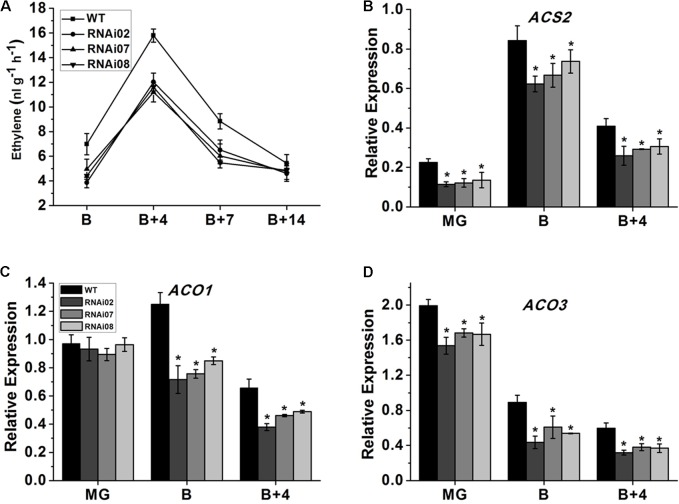
The ethylene content measurement and biosynthesis related gene expression. **(A)** The ethylene content produced by fruits of wild-type and transgenic lines. **(B–D)** Relative transcript level of genes regulating ethylene biosynthesis. Each value represents the mean ± SE of three biological replicates. Asterisks indicate a significant difference (*P* < 0.05).

Ethylene is considered to be involved in the ripening of climacteric fruit (Alexander and Grierson, 2002) and regulates the accumulation of carotenoids in tomato fruit (Maunders et al., 1987). Therefore, we measured the ethylene production of wild-type and *SlMBP15*-silenced tomato fruits at different stages. As shown in **Figure 6A**, the repression of *SlMBP15* decreased the ethylene production at the B, B + 4, and B + 7 stages.

Subsequently, we carried out qPCR to further investigate the underlying reasons for the alteration in ethylene biosynthesis. The ethylene biosynthesis-related genes *ACS* (1-aminocyclopropane-1-carboxylatesynthase)*2* (Adams and Yang, 1979), *ACO* (1-aminocyclopropane-1-carboxylate oxidase)*1* and *ACO3* (Alexander and Grierson, 2002) were detected in the pericarps of the wild-type and each transgenic line at the MG, B and B + 4 stages. As shown in **Figures 6B–D** the mRNA levels of *ACS2* and *ACO3* were distinctly lower in all stages we examined while that of *ACO1* was downregulated only in the B and B + 4 stages. In sum, *SlMBP15* downregulation repressed ethylene biosynthesis in tomato fruit.

### *SlMBP15* Influenced the Expression of Ripening Associated Genes

**FIGURE 7:**
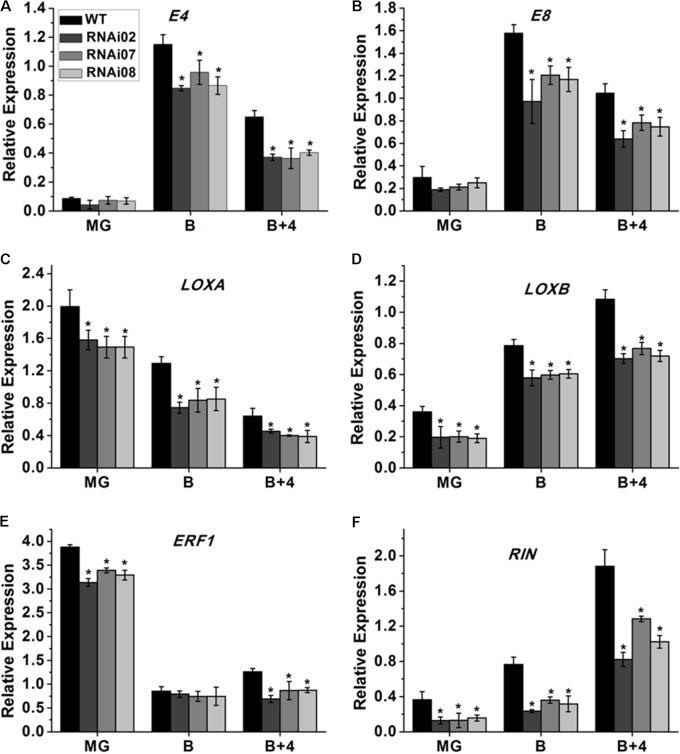
Detection of genes regulating tomato fruit ripening. **(A–F)** Relative expression of *E4*, *E8*, *LOXA*, *LOXB*, *ERF1*, and *RIN* in RNAi lines and the wild type. Each value represents the mean ± SE of three biological replicates. Asterisks indicate a significant difference (*P* < 0.05).

Tomato ripening is a complicated process that involves a series of changes in physiology and biochemistry. A large number of genes are involved alone or together to regulate the process. Some genes have been identified to be essential for tomato fruit ripening. Here, we also performed qPCR to study the expression of genes in the pericarp that modulate tomato ripening. The qPCR analysis showed that the transcription levels of *E4*, *E8* and *ERF1*, which also serve as ethylene-responsive genes (Lincoln et al., 1987; Lincoln and Fischer, 1988; Penarrubia et al., 1992), were all downregulated in the transgenic lines (**Figures 7A,B,E**). Additionally, the fruit-specific lipoxygenase genes *LOXA* and *LOXB* (Griffiths et al., 1999) were also inhibited after silencing *SlMBP15* (**Figures 7C,D**). *RIN* is a MADS-box regulator that affects the color of tomato fruit during ripening (Ito et al., 2017). In the *SlMBP15*-silenced transgenic lines, the transcript level of *RIN* was also reduced (**Figure 7F**). These results indicated that repression of *SlMBP15* has an impact on the expression of ripening associated genes.

### SlMBP15 Interacted With RIN in Yeast

**FIGURE 8:**
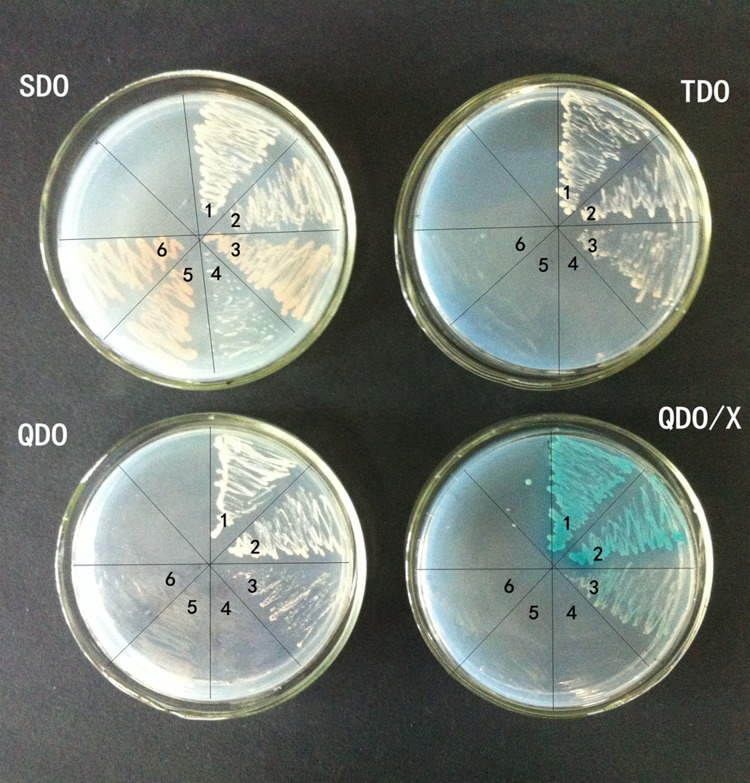
Yeast two-hybrid assay for SlMBP15 and RIN. SDO, SD medium lacking Trp; TDO, SD medium lacking Trp, His, and Ade; QDO, SD medium lacking Trp, Leu, His, and Ade; QDO/X, SD medium lacking Trp, Leu, His, and Ade with X-a-Gal. Numbered wedges are as follows: 1, pGBKT7-MBP15 and pGADT7-RIN (interaction of SlMBP15 and SlMADS-RIN); 2, pGBKT7-53 and pGADT7-T (positive control); 3, pGBKT7-Lam and pGADT7-T (negative control); 4, pGBKT7-MBP15 (autoactivation assay); 5, pGBKT7 and pGADT7-RIN (empty bait vector); 6, pGBKT7-MBP15 and pGADT7 (empty prey vector).

*RIN* is a key regulator of the ripening-gene expression network, with 100s of gene targets controlling changes in color, flavor, texture and taste during tomato fruit ripening (Fujisawa et al., 2013). Previous research suggested that MADS-box proteins usually function by interacting with each other to form higher-order complexes (Leseberg et al., 2008). In our study, the expression of the MADS-box gene *RIN* was inhibited in *SlMBP15*-silenced tomato. Therefore, we considered whether SlMBP15 interacts with RIN. To confirm this hypothesis, we performed a yeast two-hybrid assay. **Figure 8** shows that the yeast grown on DDO/X medium turned blue, as did the positive control, which indicates that SlMBP15 interacts with SlMADS-RIN in yeast.

## Discussion

Since the 1990s, many studies on the MADS-box have been carried out, and many MADS-box genes have been isolated and characterized. Until now, MADS-box genes have been proved to participate in many events during the plant life cycle. However, as a transcription factor family with more than 100 members (investigated by our laboratory, not published), only a small number of MADS-box members have been well studied. Here, we elucidated the function of a FLC/MAF clade gene, *SlMBP15*. The expression profile predicted by Multi-Plant eFP Browser 2.0^2^ showed that the expression of *SlMBP15* in fruits was higher than in other organs and that the expression in fruits after the B stages was higher than that of IMG and MG, which is consistent with our results (**Supplementary Table S5**). Recently, Shinozaki et al. (2018) showed the expression pattern of genes in individual cell and tissue types. It seems that the expression of *SlMBP15* has no change in the total pericarp during fruit development and ripening, which differed from our results (**Supplementary Figure S2**). However, the expression patterns obtain in two different ways were all detected in *cv. Heinz* while our expression pattern and wild-type tomato plant materials were from *cv. Ailsa Craig AC^++^*. This probably caused the difference in the expression of *SlMBP15*. The expression profile in the wild-type and ripening-inhibited mutants implied that *SlMBP15* may play a direct or indirect role in tomato fruit ripening (**Figures 1C,D**). Therefore, the *SlMBP15*-silenced transgenic tomato was generated for further functional study.

### *SlMBP15* Affects Plant Vegetative Growth in Tomato Plants

Previous research showed that MADS-box genes are associated with morphogenesis of almost all organs and throughout the entire plant life cycle (Guo et al., 2016). In our study, the *SlMBP15*-silenced plants were shorter than the wild-type plants, and the phenotype was further confirmed by plant height measurements (**Figures 3A–D**). It has been reported that overexpression of *SlMBP11* can also lead to reduced plant height due to a reduction in GA biosynthesis (Guo et al., 2017). Hence, we supposed that the variation in GA level may result in the dwarf phenotype in *SlMBP15*-silenced lines. To test this hypothesis, we examined the GA content in the wild-type and transgenic lines, and the result confirmed our hypothesis (**Figure 3E**). Moreover, we studied the GA biosynthesis and transduction-related genes to further explore the molecular mechanism. The qPCR result showed that GA-biosynthesis genes (*GA3ox1*, *GA3ox2*, *GA20ox1* and *GA20ox2*), a GA-induced gene (*GAST1*) and a GA-receptor gene (*GID2*) were all inhibited in the transgenic lines compared with the wild-type (**Figure 4**). These results indicated that *SlMBP15* influences the plant height and internode length in tomato by modulating GA biosynthesis and transduction. In addition, the leaves of *SlMBP15*-silenced transgenic lines were darker green than those of the wild-type, and the accumulation of chlorophyll and the transcription of *GLK1* and *GLK2* were elevated, which demonstrated that *SlMBP15* probably influenced the accumulation of chlorophyll in the leaf by regulating the chloroplast formation controlled by *GLK1* and *GLK2*.

In the introduction, we mentioned that some tomato MADS-box genes have been considered to influence different aspects of plant vegetative growth, such as leaf development, plant height, lateral organ development and AZ formation. Here, we identified SlMBP15 as a novel regulator of tomato vegetative growth via its influence on plant height and leaf color. Tomato plants overexpressing *SlMBP11* and with silenced *SlMBP15* showed the same phenotype of plant height and similar gene expression trends for GA-biosynthesis genes. These results indicate that the MADS-box gene does have an effect on the development of the tomato stem. In addition, in this regulatory process, some members function positively, while some function negatively. They cooperate to keep a balance during the development process.

### *SlMBP15* Acts as a Promoter of Tomato Fruit Ripening

It has been proved that a large amount of ethylene is required at the beginning of fruit ripening (Bleecker and Kende, 2000) and that the accumulation of carotenoids is a sign of tomato fruit ripening. In previous studies, several MADS-box genes that regulated fruit ripening were proved to affect the carotenoid accumulation and ethylene production. For example, *SlMADS1* functions as an inhibitor in tomato fruit ripening, and repression of this gene increased ethylene production and carotenoid accumulation (Dong et al., 2013). However, the pattern is the opposite in *TAGL1*-reduced tomato, in which carotenoid and ethylene content were both decreased (Vrebalov et al., 2009), which indicates that *TAGL1* acts as a promoter of tomato fruit ripening. In *SlFYFL*-overexpressing tomato, fruit ripening was delayed by inhibiting carotenoid accumulation and ethylene production (Xie et al., 2014).

In our study, the accumulation of carotenoid was significantly reduced in the pericarp of *SlMBP15*-silenced fruits at B + 4 and B + 7 compared with the wild-type (**Figure 5C**). *PSY1* is a major regulator of metabolic flux toward downstream carotenoids, which is induced by ethylene during fruit ripening (Fray and Grierson, 1993). The PDS and ZDS are two enzymes that work downstream of *PSY1*. The qPCR results revealed that the transcription of these three genes involved in carotenoid biosynthesis was all remarkably reduced in *SlMBP15*-silenced lines (**Figures 5D–F**), which revealed that *SlMBP15* regulates the biosynthesis of carotenoids. In addition, the ethylene production in the transgenic lines was also repressed. The ACS and ACO are two rate-limiting enzymes for the ethylene biosynthesis. The ACS catalyzes the conversion of SAM (*s*-adenosyl-*l*-methionine) to ACC (aminocyclopropane-1-carboxylic acid), while the ACO modulate the conversion of ACC to ethylene (Kende, 1993). It has been reported that repression of *ACS2* inhibited tomato fruit ripening (Oeller et al., 1991). Besides, *ACO1* and *ACO3* trigger fruit ripening in tomato (Alexander and Grierson, 2002). The significantly decreased expression of *ACS1*, *ACO1* and *ACO3* (**Figures 6B–D**) indicated that the silencing of *SlMBP15* suppressed the ethylene biosynthesis in fruit.

Silencing *SlMBP15* also down-regulated the transcription of ripening-associated genes including *E4*, *E8*, *LOXA*, *LOXB*, *ERF1* and *RIN*. *E4* and *E8* are well-known and important ethylene response factors involved in fruit ripening (Lincoln and Fischer, 1988). The LOXs are enzymes that catalyze the lipoxygenase pathway to transform linoleic and linolenic acids into 9-hydroperoxides and 13-hydroperoxides (Chen et al., 2004) which are involved in the volatile production that is key for tomato flavor. Moreover, LOXB is a fruit-specific lipoxygenase that is induced by ethylene (Griffiths et al., 1999). *ERF1* is an ethylene-responsive factor, while *RIN* is a MADS-box gene that affects the accumulation of carotenoids in tomato (Ito et al., 2017). The expression level of these genes was reduced in the *SlMBP15*-silenced lines. Taken together, these results indicated that *SlMBP15* acts as a promoter of fruit ripening. However, owing to the complex process of fruit ripening controlled by MADS-box genes, more experiments are needed to figure out whether *SlMBP15* plays direct or indirect roles in tomato fruit ripening.

RIN was considered as a major regulator that is essential for the induction of ripening, although recent research indicated that *RIN* does not play such a crucial role in fruit ripening and that the substantial inhibition of fruit ripening due to the *rin* mutation is caused by the gain-of-function of RIN-MC chimera (Ito et al., 2017); it is true that *RIN* modulates ethylene synthesis, inhibits fruit coloring and regulates the expression of many ripening-associated genes (Lin et al., 2008; Martel et al., 2011; Qin et al., 2012; Ito et al., 2017). MADS-box proteins usually function through the formation of heterodimers, homodimers, or higher-order complexes with each other (Favaro et al., 2002) to perform diverse functions. In this study, the ethylene production in tomato fruit was downregulated, and the fruit color turned lighter in the *SlMBP15*-silenced lines. Moreover, *SlMBP15* regulated the expression of ripening-associated genes. Therefore, we carried out a yeast two-hybrid assay to examine whether *SlMBP15* interacts with RIN. The result provided evidence for the interaction between SlMBP15 and RIN. In the previous study, some genes that mediate tomato fruit ripening were proved to interact with RIN such as *SlMADS1* (Dong et al., 2013), *SlFYFL* (Xie et al., 2014), *FUL1*, *FUL2* (Leseberg et al., 2008), *TAGL1* (Martel et al., 2011) and *SlMBP8* (Yin et al., 2017a). We conclude that SlMBP15 may take part in regulating the expression of carotenoid biosynthesis, ethylene biosynthesis and ripening-associated genes by cooperating with RIN.

The results showed that the MADS-box contributes substantially to the complex regulatory process of tomato fruit ripening. MADS-box genes participate in fruit ripening, but the roles they play and the positions they occupy in the regulatory network are different. Some MADS-box genes promote tomato fruit ripening, while some inhibit it. In the study of *SlMBP15*, it is interesting that the other FLC/MAF clade gene, *SlMBP8*, which is highly homologous to *SlMBP15*, is also involved in tomato fruit ripening (Yin et al., 2017a); however, unlike *SlMBP15*, *SlMBP8* inhibits ripening. We speculated that *SlMB15* may have been produced by *SlMBP8* duplication and divergence, or vice versa and they probably work in opposite directions in the same regulatory pathway.

In this study, we identified a novel fruit ripening promoter that belongs to the MADS-box family. *SlMBP15* regulates the transcription of genes involved in carotenoid and ethylene biosynthesis. *SlMBP15* also affects the biosynthesis of chlorophyll and GA. Its function in tomato fruit ripening has been revealed here, but the higher-level developmental regulatory mechanism remains to be discovered. Since repression of *SlMBP15* delays fruit ripening, it may be beneficial for extending the shelf life of tomato, which is a character with application potential.

## Author Contributions

GC and ZH designed and managed the research work and improved the manuscript. WY, BT, YW, CL, and YZ performed the experiments. XY wrote the manuscript.

## Conflict of Interest Statement

The authors declare that the research was conducted in the absence of any commercial or financial relationships that could be construed as a potential conflict of interest. The reviewer JB and handling Editor declared their shared affiliation.

## Footnotes

1 https://blast.ncbi.nlm.nih.gov/Blast.cgi

2 http://bar.utoronto.ca/efp2/

## Supplementary Material

FIGURE S1 Alignment of the protein sequences of SlMBP15 and the transcript variants.

FIGURE S2 The predicted expression profile of *SlMBP15* in tissues and cells in tomato fruit.

TABLE S1 Gene information for *SlMBP15*.

TABLE S2 The accession numbers of proteins contained in multiple sequence alignment and phylogenic analysis.

TABLE S3 Primers used for Quantitative PCR analysis.

TABLE S4 Accession numbers of *SlMBP15* and the transcript variants.

TABLE S5 Predicted expression profile of *SlMBP15*.
