# Supplementary figures and images for: Suppression of SlMBP15 Inhibits Plant Vegetative Growth and Delays Fruit Ripening in Tomato
Source: Front Plant Sci. 2018 Jul 4;9:938. doi: 10.3389/fpls.2018.00938 (PMC6039764; doi:10.3389/fpls.2018.00938)

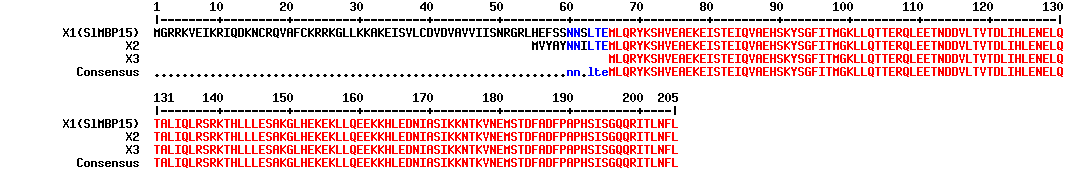

Supplement: FIGURE S1 — Alignment of the protein sequences of SlMBP15 and the transcript variants. [file Image_1.TIF]

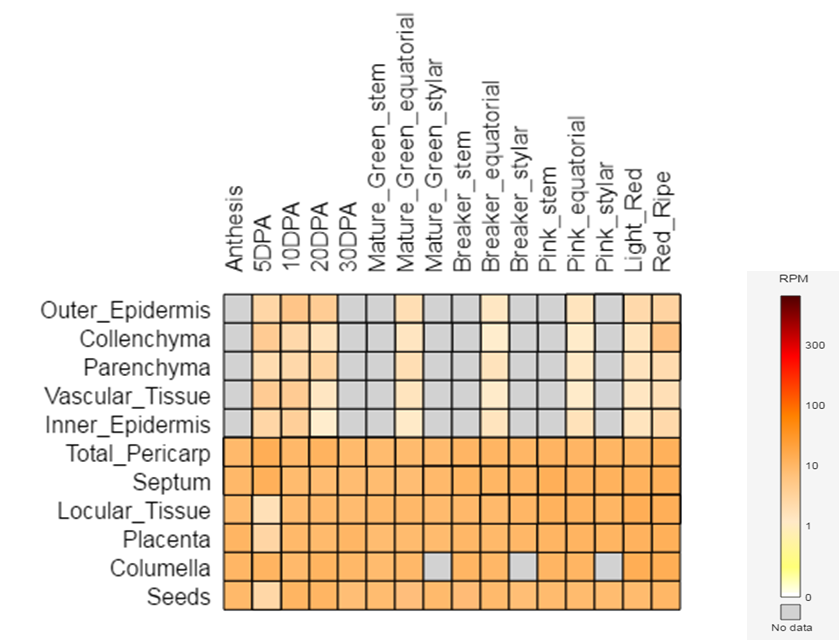

Supplement: FIGURE S2 — The predicted expression profile of SlMBP15 in tissues and cells in tomato fruit. [file Image_2.TIF]
