# Supplementary material for: Suppression of SlMBP15 Inhibits Plant Vegetative Growth and Delays Fruit Ripening in Tomato
Source: Front Plant Sci. 2018 Jul 4;9:938. doi: 10.3389/fpls.2018.00938 (PMC6039764; doi:10.3389/fpls.2018.00938)
Supplement: TABLE S1 — Gene information for SlMBP15. [file Table_1.DOCX]

**Gene information for *SlMBP15***

**Accession number:**

XM_004252663 (NCBI)

Solyc12g087810.2 (SGN)

**Link:** <https://www.ncbi.nlm.nih.gov/nucleotide/XM_004252663.3?report=genbank&log$=nucltop&blast_rank=1&RID=CSAKVBYM014> (NCBI)

<https://solgenomics.net/tools/blast/show_match_seq.pl?blast_db_id=297;id=Solyc12g087810.2;hilite_coords=81-516> (SGN)

**Full cDNA sequence (ORF=618 bp):**

AAATATGCCAAGTAAAAAACAGAGAGGGAAGACGAAAAGGGACCGAAGAGATTTAATTAGAGTGAAGTGAGTAAAAGAACAAAAATGGTAACTCTGTAACAAGCGCACACACAAAAGAGAAGAGCTGGGTAGGTGTAACGCCGATGATAGAAGAATCTTCTAGACATCTGAAATTTGTGGATTAGGGCTTTTCATCGGCGAAAAAAAAAATGGGGCGAAGGAAGGTAGAAATTAAGCGAATTCAAGATAAAAATTGCAGGCAAGTTGCGTTCTGTAAACGGAGGAAAGGTTTATTGAAGAAAGCTAAAGAAATTTCCGTTCTCTGCGATGTCGATGTTGCTGTTGTTATCATCTCAAATCGAGGCAGGCTCCATGAATTCTCCAGCAATAACAGTCTGACAGAAATGCTTCAACGATACAAAAGCCACGTCGAAGCAGAAAAAGAGATCTCTACAGAAATCCAGGTGGCAGAGCACTCTAAATACTCAGGGTTCATAACAATGGGAAAACTGCTACAAACAACAGAAAGGCAACTCGAGGAAACTAATGATGATGTTCTCACTGTGACTGACCTTATCCATTTGGAGAACGAACTTCAAACTGCTCTAATACAACTCAGATCTAGAAAGACACATTTGTTGCTTGAATCTGCTAAGGGTCTTCATGAGAAGGAAAAACTGCTGCAAGAGGAAAAGAAACATCTGGAGGACAATATAGCTAGTATCAAGAAAAACACAAAAGTGAATGAGATGTCTACGGATTTCGCTGACTTTCCAGCACCCCATTCGATTTCTGGACAACAGAGAATAACCCTGAATTTCCTCTAGTACTTGTCGAATAGCCCCAGGGGAATAACCTGTGAAATGAGCCAGAGTCGAGATGCATCTGGAGATAACTAATATTGTGCAGCTTCTATAGAACTAATTATTGTTTCTATTTTGATGATGAACATTTAGATTTGTACAATCACTTTGTTGGGAACATTTACTTCAAACAAAAATATTGTAAATTGGTTTTGACAAGCCTAATTTTCCATCTTTTTCTGAGTGAAA

**Full protein sequence (205 aa):**

MGRRKVEIKRIQDKNCRQVAFCKRRKGLLKKAKEISVLCDVDVAVVIISNRGRLHEFSSNNSLTEMLQRYKSHVEAEKEISTEIQVAEHSKYSGFITMGKLLQTTERQLEETNDDVLTVTDLIHLENELQTALIQLRSRKTHLLLESAKGLHEKEKLLQEEKKHLEDNIASIKKNTKVNEMSTDFADFPAPHSISGQQRITLNFL
