# Supplementary material for: Suppression of SlMBP15 Inhibits Plant Vegetative Growth and Delays Fruit Ripening in Tomato
Source: Front Plant Sci. 2018 Jul 4;9:938. doi: 10.3389/fpls.2018.00938 (PMC6039764; doi:10.3389/fpls.2018.00938)
Supplement: TABLE S2 — The accession numbers of proteins contained in multiple sequence alignment and phylogenic analysis. [file Table_2.DOCX]

**Table S2. The accession numbers of proteins contained in multiple sequence alignment and phylogenic analysis**

| Protein name | Accession number |
| --- | --- |
| TDR4 | NP_001234173.2 |
| MADS-MC | NP_001234665.1 |
| MADS-RIN | NP_001234670.1 |
| FLC | AFU51419.1 |
| FLC1 | BAD42943.1 |
| MAF2 | ACL93422.1 |
| AtMAF3 | ACL93439.1 |
| AtMAF4 | ACL93428.1 |
| AtMAF5 | ACL93400.1 |
| SlMBP8 | XP_004252712.1 |
| SlMBP15 | XP_010314580.1 |
| SlMBP25 | XP_004239235.1 |
