# Supplementary material for: Suppression of SlMBP15 Inhibits Plant Vegetative Growth and Delays Fruit Ripening in Tomato
Source: Front Plant Sci. 2018 Jul 4;9:938. doi: 10.3389/fpls.2018.00938 (PMC6039764; doi:10.3389/fpls.2018.00938)
Supplement: TABLE S3 — Primers used for Quantitative PCR analysis. [file Table_3.DOCX]

**Table S3. Primers used for Quantitative PCR analysis**

| Gene | Primer sequence: (5ˊ-3ˊ) | Accession Number |
| --- | --- | --- |
| *SlCAC*-Q-F  *SlCAC*-Q-R | CCTCCGTTGTGATGTAACTGG  ATTGGTGGAAAGTAACATCATCG | XM-004244491 |
| *SlMBP15*-Q-F  *SlMBP15*-Q-F | GGCAGAGCACTCTAAAGACTC  GGAAATTCAGGGTTATTCTC | XM_004252663 |
| *DCL*-Q-F | CCGCAAGGATGGTGAAACA | U55219 |
| *DCL*-Q-R | TCCGCTTCCGAAAATGCC |  |
| *GLK1*-Q-F | GAATTTTCCGTAAGCAGTGGTG | JQ316460 |
| *GLK1*-Q-R | CTTCTCCTTGATTTAGGCTCGT |  |
| *GLK2*-Q-F | ACAATCGGAGGCGGAGGA | JQ316459 |
| *GLK2*-Q-R | CAAGGAGTGCCTGGTACAAGAG |  |
| *ACO1-*Q-F  *ACO1-*Q*-*R | ACAAACAGACGGGACACGAA  CTCTTTGGCTTGAAACTTGA | NM_001247095.1 |
| *ACO3-*Q*-*F  *ACO3-*Q*-*R | CAAGCAAGTTTATCCGAAAT  CATTAGCTTCCATAGCCTTC | Z54199 |
| *ACS2*-Q-F  *ACS2*-Q-R | GAAAGAGTTGTTATGGCTGGTG  GCTGGGTAGTATGGTGAAGGT | AY3269 |
| *E4*-Q-F  *E4*-Q-R | AGGGTAACAACAGCAGTAGCA  CCCAACCTCCGTCTTCAC | S44898.1 |
| *E8*-Q-F  *E8*-Q-R | GGCACCATTCAACATACCG  CTTTCACCGAAGAAGCACG | X13437.1 |
| *ERF1-Q-F*  *ERF1-Q-R* | TTTTAGTATCGGATGGACG  GGCGGAGAAACAGAAGTA | NM_001247919.1 |
| *RIN-Q-F*  *RIN-Q-R* | GGAACCCAAACTTCATCAGA  TTGTCCCAAATCCTCACCTA | NM_001247741.1 |
| *PSY1-Q-F*  *PSY1-Q-R* | AGAGGTGGTGGAAAGCAA  TCTCGGGAGTCATTAGCAT | EF157835 |
| *LoxA*-Q-F  *LoxA*-Q-R | GAGGCGTGGGATAGGA  GGATACGGGTAGTCAGCA | U09026.1 |
| *LoxB*-Q-F  *LoxB*-Q-R | TGCTACAATGACTTGGGTGAA  CCTGTCCTGCCTCTACG | U09025.1 |
| *PDS-Q-F*  *PDS-Q-R* | GCTTTACCCGCTCCTTTA  ACCTTGCTTTCTCATCCA | S36691.1 |
| *ZDS*-Q-F  *ZDS*-Q-R | GGTGGGTGCTGAAAAAAAT  GGAAAGCGGAAATCAAGTT | NM_001247454.1 |
| *GA20ox1*-Q-F  *GA20ox1*-Q-R | TTCTCAAATTGGCTTCATGATCAA  TTCCCCCTAATTCCCATAACAT | NM_001247141.1 |
| *GA20ox2*-Q-F  *GA20ox2*-Q-R | TAAGAAGGATAAGGTGGTGAGGC  CCGTAGTTTTCTGTTGAAGCCA | NM_124560.4 |
| *GA3ox1*-Q-F  *GA3ox1*-Q-R | ATAGGCACCCACCCTTGTATA  GGATGAAAGTGCCTTGTCAAAAT | NM_101424.3 |
| *GA3ox2*-Q-F  *GA3ox2*-Q-R | GTAGACCAAAGGAACCCTCAAAT  GCCGAACAGATGAAAGTGCT | [NM_106683.2](https://www.ncbi.nlm.nih.gov/nucleotide/1063693619?report=genbank&log$=nucltop&blast_rank=1&RID=5MEYZXSV013) |
| *GID1*-Q-F  *GID1*-Q-R | GCGGTGTTGTTGAATGAGAATC  GTCTTGTGCAGATCAGCTCCC | NM_001347058.1 |
| *GAST1*-Q-F  *GAST1*-Q-R | CAACAACAGAGAAATAACCAAC  TTATACGATGTCTTTGAACACC | NM_001309377.1 |
