# Supplementary material for: Suppression of SlMBP15 Inhibits Plant Vegetative Growth and Delays Fruit Ripening in Tomato
Source: Front Plant Sci. 2018 Jul 4;9:938. doi: 10.3389/fpls.2018.00938 (PMC6039764; doi:10.3389/fpls.2018.00938)
Supplement: TABLE S4 — Accession numbers of SlMBP15 and the transcript variants. [file Table_4.DOCX]

**Table S4. Accession numbers of *SlMBP15* and the transcript variants**

| Name | Accession number |
| --- | --- |
| Transcript variant X1 (*SlMBP15*) | XM_004252663.3 |
| Transcript variant X2 | XM_010316278.2 |
| Transcript variant X3 | XM_010316279.2 |
| Transcript variant X4 | XM_010316280.2 |
