# Supplementary material for: Suppression of SlMBP15 Inhibits Plant Vegetative Growth and Delays Fruit Ripening in Tomato
Source: Front Plant Sci. 2018 Jul 4;9:938. doi: 10.3389/fpls.2018.00938 (PMC6039764; doi:10.3389/fpls.2018.00938)
Supplement: TABLE S5 — Predicted expression profile of SlMBP15. [file Table_5.DOCX]

**Table S5. Predicted expression profile of *SlMBP15***

| Variety | Rt | L | F(un-open) | F | Stages | | | | | |
| --- | --- | --- | --- | --- | --- | --- | --- | --- | --- | --- |
|  |  |  |  |  | 1 cm | 2cm | 3cm | MG | B | B+10 |
| Heinz | 6.3 | 21.9 | 24.49 | 23.07 | 25.49 | 31.15 | 34.49 | 25.43 | 38.1 | 43.68 |

***Data was obtained from http://bar.utoronto.ca/efp2/.**
